# Supplementary figures and images for: Dopamine Receptor-Expressing Neurons Are Differently Distributed throughout Layers of the Motor Cortex to Control Dexterity
Source: eNeuro. 2024 Mar 15;11(3):ENEURO.0490-23.2023. doi: 10.1523/ENEURO.0490-23.2023 (PMC10965237; doi:10.1523/ENEURO.0490-23.2023)

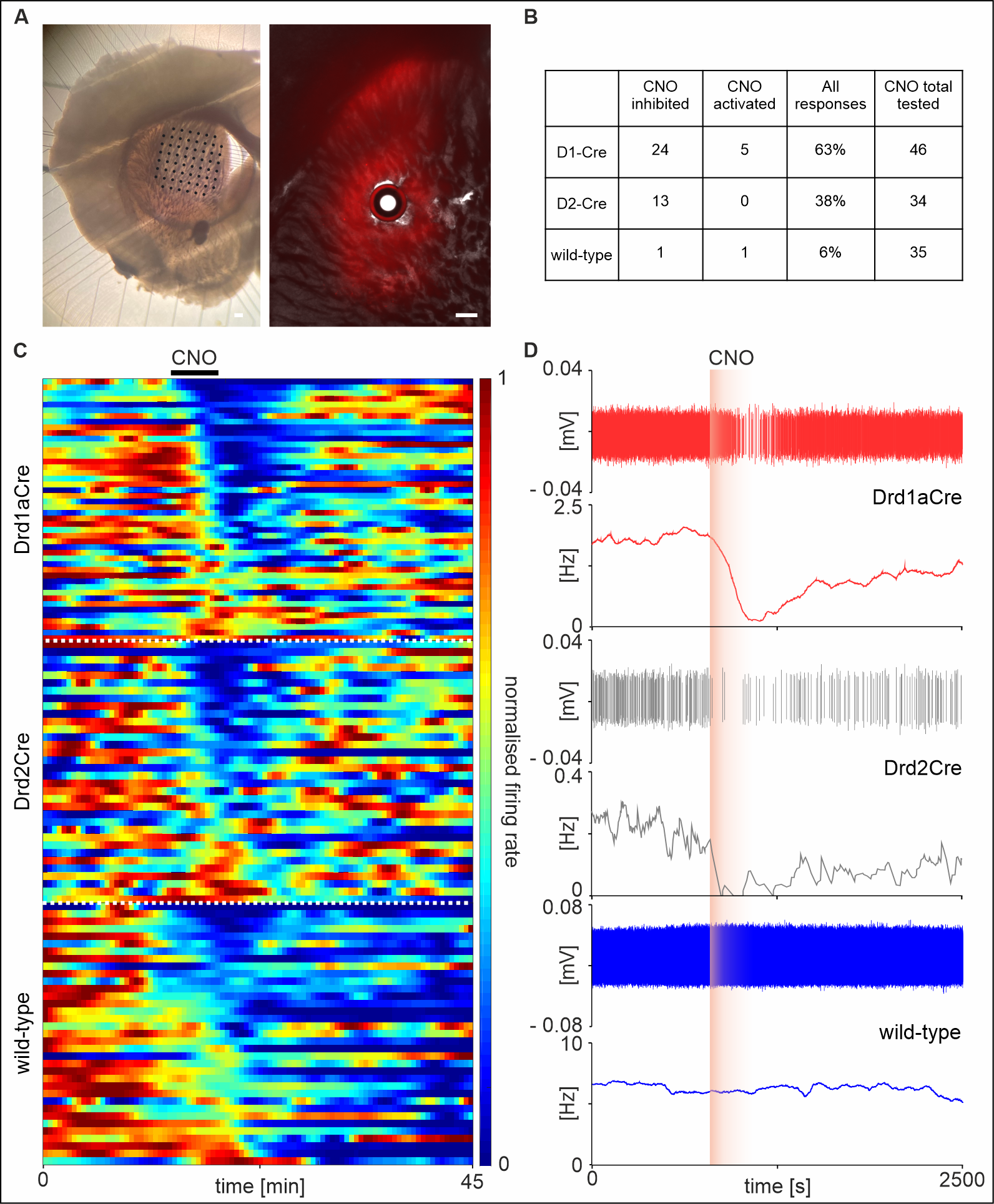

Supplement: Figure 6-1 — Validation of chemogenetic inhibition of neuronal activity in Drd1aCre and Drd2Cre mice using striatal neurons as an example. (A) Coronal section obtained from a wild-type (left) and Drd1aCre mouse (right) showing slice placement upon the multi-electrode array (black dots indicate single recording electrodes) and hM4Di-mCherry expression in the striatum after recording. Scale bars, 200 μm. (B) Table summarizing recorded neurons and their responses to CNO application. (C) Temporal heatmaps encoding single-unit activity (SUA) of all recorded neurons and their response to CNO application (10 μM, 10 ml; indicated by a black line). Each row indicates a single unit. White dotted horizontal lines classify units to either Drd1aCre mice (n = 46 units), Drd2Cre mice (n = 34), or wild-type mice (n = 35). The cell activity was normalized from 0 to 1 and was sorted by the defined window corresponding to the CNO response. Bin, 30 s. (D) Examples of neurons recorded during CNO administration in Drd1aCre, Drd2Cre and wild-type mice. Top panels show separated spikes of a single neuron, bottom panels show a corresponding frequency histogram. The orange-shaded rectangle indicates the duration of CNO action. Bin, 60 s. (B-D) Slices were obtained from n = 1 hM4Di-mCherry injected animal for each group, n = 4 slices per animal. Download Figure 6-1, TIF file. [file eneuro-11-ENEURO.0490-23.2023-s002.tif]
